# Responses of growth and quality of *Glycyrrhiza uralensis* to salt-alkali-tolerant microbial inoculant via the remodeling of rhizosphere microenvironments in moderate and severe saline-alkali soils

**DOI:** 10.1186/s40793-026-00941-4

**Published:** 2026-08-13

**Authors:** Qi Guo, Jun Zhang, Xin Li, Peiyao Pei, Peiya Wang, Hui Yang, Xian Xue

**Affiliations:** 1 Gansu International Science and Technology Cooperation Base of Microorganism and Plant Germplasm Resources & Genetic Improvement, Institute of Biology, Gansu Academy of Sciences, Lanzhou, China; 2 Key Laboratory of Desert and Desertification, Northwest Institute of Eco- Environment and Resources, Chinese Academy of Sciences, Lanzhou, China

**Keywords:** *Glycyrrhiza uralensis*, Salt-alkali-tolerant microbial inoculant, Saline-alkali soils, Direct seeding vs. seedling transplanting, Root growth, Bioactive compounds, Rhizosphere soil nutrients, Microbial community diversity

## Abstract

*Glycyrrhiza uralensis* is an ecologically and economically important medicinal species for saline-alkali land restoration in arid northwest China. Nevertheless, excessive soil salinity, alkalinity and nutrient deficiency substantially restrict its growth and degrade medicinal quality. Microbial inoculation serves as a promising strategy to alleviate salt-alkali stress, yet the field stability and rhizosphere regulatory mechanisms of inoculants under diverse cultivation regimes remain poorly understood, limiting their field application. Building on our prior strain screening and preliminary field validation, this study utilized a composite inoculant containing *Pseudomonas silesiensis*, *Arthrobacter* sp. GCG3 and *Rhizobium* sp. DG1, and investigated its effects on *G. uralensis* growth, bioactive metabolites, rhizosphere soil properties and microbial communities under three field viable cultivation scenarios (direct seeding and seedling transplanting in moderately saline-alkali soil, seedling transplanting in severely saline-alkali soil) with respective tailored fertilization, planting densities and inoculation schedules. The results revealed that under all scenarios, the inoculation induced an increasing trend in the root dry weight and bioactive compound accumulation, with markedly higher dry root weight in seedling transplanting scenarios (101.68%, moderately saline-alkali soil; 53.96%, severely saline-alkali soil, *P* < 0.05). Glycyrrhizic acid contents per plant rose by 57.32, 106.11 and 6.56%, while those of liquiritin rose by 42.50, 177.24 and 42.34%, respectively. The inoculant barely altered rhizosphere pH and soluble salt contents, yet universally regulated rhizosphere nutrient pools, which were reflected in a uniform reduction of nitrate nitrogen and scenario-specific shifts in other available nutrients as well as soil organic matter. High-throughput sequencing verified that this inoculant could reshape the rhizosphere microbial community structure of *G. uralensis*; across all scenarios, the relative abundance of pathogenic *Fusarium* significantly decreased, and indigenous beneficial bacteria and fungi were enriched. This inoculant exhibited stable growth-promoting effects across all cultivation regimes. The combined application of microbial inoculation and seedling transplanting is therefore recommended for large-scale and high-quality cultivation of *G. uralensis* in moderately saline-alkali soils of arid northwestern China, as this integrated practice maximizes plant growth, medicinal compound accumulation, and rhizosphere microenvironment optimization.

## Introduction

Salinization of agricultural fields is a global crisis that has become a key issue restricting sustainable regional agricultural development and ecological security [63, 83, 117]. Due to the arid climate and intense evaporation [53, 57], alongside irrational irrigation practices [28, 55], soil salinization is particularly severe in the arid regions of northwest China. In the region, Gansu Province has over 2.1 million hectares of saline-alkali cultivated land and wasteland, mainly distributed in the Hexi Corridor and the Yellow River Irrigation District [40]. Salt stress restrains plant development [12, 100] and causes land degradation [36, 76] and biodiversity loss [41, 80], greatly hindering local ecological rehabilitation and arable land quality improvement.

For the amelioration of saline-alkali soils and sustainable resource utilization, the perennial salt-tolerant medicinal legume *Glycyrrhiza uralensis* exhibits prominent advantages [81]. It can be infected by rhizobia to form nitrogen-fixing root nodules, which convert atmospheric nitrogen into bioavailable nitrogen, enriching soil nitrogen pools [32, 73, 98]. Simultaneously, its extensive root system can promote soil aggregation, alleviate topsoil salt accumulation, and gradually improve aeration, water-holding capacity and nutrientretention capacity of saline-alkali soils [56, 62, 110]. The roots of *G. uralensis* are also rich in the bioactive components glycyrrhizic acid and liquiritin [46, 47], featuring anti-inflammatory [15, 51], hepatoprotective [31, 120], antiviral [5, 38], and anticancer [78] effects. These root metabolites are highly sought-after in the pharmaceutical and food industries [82, 93]. Therefore, *G. uralensis* is an ideal crop for balancing ecological restoration and industrial development. Nevertheless, all salt-tolerant plants possess a salt-alkali tolerance threshold. Once soil salinity and alkalinity exceed this value, plant growth is suppressed [111, 112], which greatly restricts the large-scale cultivation of *G. uralensis* in moderately and severely saline-alkali soils.

Microbial inoculant technology that has emerged in recent years provides a promising strategy to break the salt-tolerance limitation of *G. uralensis*. Currently, the most widely used microbes are plant growth-promoting rhizobacteria (PGPR), which inhabit the rhizosphere and promote plant growth and productivity through multiple mechanisms [66, 101]. Salt-alkali-tolerant PGPR are screened from the rhizospheres of salt-tolerant plants and halophytes under extreme salt stress. Possessing unique metabolic mechanisms and stress tolerance potentials, they survive in saline soils and efficiently promote plant growth with high application success. Khalilpour et al. [50], Kumar [54], Misra [71].

However, the effectiveness of microbial inoculants may be strongly heterogeneous across different cropping systems [14], limiting their broad application. For instance, inoculants performing well in slightly saline-alkali soil may show a sharp decline in efficacy in severely saline-alkali soil [67]. Inoculation approaches including seed coating and seedling dipping modulate the rhizosphere colonization capacity of inoculant strains [21]. Furthermore, inoculant efficiency varies with fertilization regimes [14], planting density [104, 105], and application frequency [79].

In our previous research, the most beneficial salt-alkali-tolerant inoculant was identified by investigating its effects on the growth performance and bioactive compound accumulation of *G. uralensis* in moderately saline-alkali soils [122]. Based on these preliminary results, we applied the inoculant to *G. uralensis* and conducted field experiments in Lanzhou, Gansu Province covering four combined scenarios: two cultivation modes (direct seeding and seedling transplanting) in moderate and severe saline-alkali soils, with distinct fertilization regimes, planting densities, and inoculation frequencies. This study aimed to elucidate the multi-scenario effects of the inoculant on *G. uralensis* growth and biomass accumulation, the accumulation of the bioactive substances glycyrrhizic acid and liquiritin, as well as rhizosphere soil nutrient contents and the diversity of rhizosphere microbial communities, and clarify the stability and efficacy of the inoculant across different scenarios. This work enriches the theory of microbial inoculant application in saline-alkali medicinal herb cultivation and provides technical guidance for large-scale planting of *G. uralensis* on analogous saline-alkali lands in northwest China.

## Materials and methods

### Study site description

Field trials were conducted in moderately and severely saline-alkali soils in Xicha Town (36°42′ N, 104°19′ E) and Heishi Town (36°50′ N, 104°31′ E), respectively, Gaolan County, Lanzhou City, Gansu Province, China. Both sites are in the temperate semi-arid continental climate zone. According to the regional climate background data of Gaolan County [116], the county’s average annual temperature is 7.2 °C, annual precipitation is 266 mm, annual evaporation is 1,660 mm, annual sunshine duration is 2768 h, and the frost-free period is 144 d. Owing to topographical differences, there are slight differences in local climate conditions between the two towns. Xicha Town is in the central plain of the Qinwangchuan fault basin in northwestern Gaolan County, with an average altitude of approximately 1,900 m. The flat and open terrain is part of the core area of the Yindaruqin Irrigation District, with local air humidity slightly higher than that in surrounding areas. Heishi Town is in northern Gaolan County in a hilly and gully area of the Loess Plateau, with an average altitude of approximately 1,800 m and relatively higher aridity.

Topsoil (0–20 cm) properties of the field in Xicha Town after deep plowing were as follows: organic matter (OM) 6.84 g kg^−1^, total nitrogen (TN) 220 mg kg^−1^, available phosphorus (AP) 41.80 mg kg^−1^, available potassium (AK) 227.22 mg kg^−1^, nitrate nitrogen (NO_3_^−^-N) 48.02 µg g^−1^, pH 7.80, and soluble salt (SS) 4.08 g kg^−1^ (7.38 g kg^−1^ before deep plowing). Given that the soil after deep plowing was the actual plant growth substrate, the soil SS content after deep plowing was used as the basis for determining the salinization level, and the Xicha field was classified as moderately salinized [22].

The topsoil (0–20 cm) properties of the field in Heishi Town were the following: 5.70 g kg^−1^ OM, 192.26 mg kg^−1^ TN, 6.80 mg kg^−1^ AP, 319.02 mg kg^−1^ AK, NO_3_^−^-N 16.12 µg g^−1^, pH 7.80, and 6.15 g kg^−1^ SS (9.56 g kg^−1^ SS before deep plowing), classifying the field as severely salinized.

### Field trial design

Trials at two experimental sites were conducted simultaneously, beginning in April 2023 and concluding at the end of September 2024. Two planting patterns were used for *G. uralensis* cultivation, including seed sowing and seedling transplanting. Seeds (harvested in 2022, with pretested germination rate exceeding 90% based on petri dish germination paper method) and one-year-old seedlings (root length: 40–45 cm, diameter: 0.8–1.0 cm, with uniform growth and free of diseases and pests) were both provided by Mingqin Gende Root Chinese Medicinal Materials Co., Ltd., Wuwei, China.

All pretreatments of *G. uralensis* seeds and seedlings were conducted at 22–25 °C in indirect sunlight. Seeds were immersed in 98% H_2_SO_4_, with the volume sufficient to fully submerge all seeds, stirred continuously for 10 min, rinsed thoroughly with tap water for 20 min, then soaked separately in either the liquid inoculant or control (local irrigation water) for 5–6 h, drained, and shade-dried until all surface water evaporated. Seedlings were disinfected by soaking for 10–15 min in 800–1000-fold dilution of 50% carbendazim wettable powder (Shandong Chengxin Biotechnology Co., Ltd., Weifang, China) prepared with local irrigation water. After draining excess moisture, the seedlings were divided into two bundles for coating with treatment or control slurry, respectively. Treatment and control slurries were separately prepared using air-dried topsoil from corresponding experimental plot. The soil was mixed with either a 100-fold diluted liquid inoculant or an equal volume of control (irrigation water), respectively, at a 1:1 volume ratio of soil to liquid.

The salt-alkali-tolerant microbial liquid inoculant was developed based on screening in previous research and composed of *Pseudomonas silesiensis*, *Arthrobacter* sp. GCG3, and *Rhizobium* sp. DG1 [122]. Each strain was separately cultivated in LB liquid medium at 28 °C, 180 r min^−1^ shaking until OD_600_ reached 0.6–0.8 (the logarithmic phase) to maintain uniform viable cell density. Each single-strain culture cell was collected by centrifugation (6000 r min^−1^, 10 min, 4 °C), resuspended in sterile water, and mixed in equal volumes. The compound suspension was standardized to 2 × 10^10^ CFU mL^−1^, stored at 4 ℃ and used within 24 h.

Four independent trial scenarios were established without cross-scenario interaction analysis. Direct seeding and seedling transplanting shared one fertilization regime in moderately saline-alkali soil, whereas a distinct fertilization strategy was used for both cultivation patterns in severely saline-alkali soil.

For the moderately salinized field trials, one month before planting, fully decomposed cow manure (1.5% N content) was uniformly applied to the soil surface at the rate of 30,000 kg ha^−1^ (corresponding to 450 kg N ha^−1^). The soil was subsequently plowed to a depth of 25–30 cm. Simultaneously, a mixed fertilizer consisting of superphosphate (300 kg ha^−1^), diammonium phosphate (225 kg ha^−1^), and potassium fulvate (75 kg ha^−1^) (Yunnan Ruifeng Chemical Co., Ltd., Kunming, China) was evenly broadcast onto the soil and incorporated through a second plowing to ensure complete homogenization with the native soil. The land was then leveled for uniform planting. For the severely salinized field trials, one month before planting, fully decomposed pig manure (2% N content) was uniformly applied to the soil surface at the rate of 70,000 kg ha^−1^ (corresponding to 1400 kg N ha^−1^). The soil was subsequently plowed to a depth of 25–30 cm. The land was subsequently leveled for uniform planting.

Each scenario adopted a randomized complete block design and contained two groups, the salt-tolerant microbial inoculant group (MI) and the control group (CK), with five replicate plots set for each group. The application rate of the liquid microbial inoculant was uniformly set at 3 mL m^−2^, based on results from our preliminary experiments. The CK group received local irrigation water as the blank control. Both treatments were administered via soil drenching around the plant roots.

For direct seeding, a flat-bed, plastic film-mulching, hole-sowing technique was adopted, with identical plot layouts for moderately and severely salinized soils. Holes (diameter: 3–4 cm) were punched in the plastic film directly above each seedling, approximately 30 d after sowing. Seedlings at 3–5 cm height were thinned to retain 1–2 vigorous individuals per hole. Seedlings were transplanted via flat-bed furrow cultivation. Guard rows matching each plot in width, cultivar and field management were arranged on all four sides of all direct-seeding and transplanting plots. Furrows were backfilled, compacted and irrigated with root-setting water right after transplanting. Detailed trial parameters for each treatment across the four scenarios are provided in Table 1.

**Table 1:** Experimental parameters of each treatment across four independent cultivation scenarios

Trial scenario	Plot length × width (m)	Plot area (m^2^)	Cultivation layout and parameters	Total for 5 replicate plots	Inoculation frequency
DS(M)	0.5 × 0.5	0.25	3 rows per plot (15 cm row spacing), with 6 holes per row (10 cm hole spacing, 2 cm hole depth). 5 seeds were sown per hole. The total sowing quantity per plot was 90 seeds.	450	4
DS(S)	0.5 × 0.5	0.25	Same as DS(M)	450	8
ST(M)	9.0 × 1.0	9.0	2 furrows per plot (15–20 cm furrow depth, 50 cm furrow spacing). 30 seedlings were placed horizontally per furrow at with a uniform 30-cm apex-to-apex spacing between adjacent seedlings. The total transplanted per plot was 60 seedlings.	300 seedlings	4
ST(S)	18.0 × 0.5	9.0	1 furrow per plot (15–20 cm furrow depth) with a plant spacing of 45 cm. The seedling placement method was consistent with that of the ST(M) scenario. The total transplanted per plot was 40 seedlings.	200 seedlings	8

DS: direct seeding; ST: seedling transplanting; M: moderately saline soil; S: severely salinized soil.

### Determination of indicators and methods

#### Determination of growth indicators and active ingredients of *G. uralensis*

After harvesting *G. uralensis*, 15 plants were randomly sampled from each replicate plot under each scenario, resulting in 75 plants for both the MI and CK groups respectively. Roots were intactly excavated, and taproot length (TRL), taproot diameter (TRD), and number of lateral roots (LRN; only primary lateral roots longer than 1 cm were counted; fine fibrous rootlets were excluded) were recorded. Subsequently, root tissues were rinsed thoroughly, air-dried at room temperature to remove surface moisture, sliced into thin sections, and oven-dried at 40 °C to constant weight for determination of the single-plant dry weight (DW). Glycyrrhizic acid and liquiritin, the characteristic bioactive constituents of *G. uralensis* roots, were quantified via the high-performance liquid chromatography (HPLC) specified in the Pharmacopoeia of the People’s Republic of China Vol. I [25], using an Agilent 1260 HPLC system (Agilent Technologies, Beijing, China). Contents of glycyrrhizic acid (GA DW) and liquiritin (LIQ DW) per plant were calculated based on the dry weight per plant and their respective mass percentages.

#### Determination of rhizosphere soil chemical properties

Rhizosphere soil refers to soil tightly attached to excavated intact *G. uralensis* roots. Roots were gently shaken to remove loosely attached soil and the adhering soil that remained was brushed into sterile bags with sterile brushes. Part of the soil was air-dried at room temperature and sieved through a 2-mm sieve, then pH, SS, OM, TN, AP, NO_3_^−^-N, and AK were determined following the methods described by Bao [11]. The remaining soil was stored at −80 °C for high-throughput sequencing of rhizosphere microbiomes.

#### High-throughput sequencing of rhizosphere soil microorganisms

Total soil microbial DNA was extracted with the FastDNA^®^ Spin Kit for Soil (MP Biomedicals, USA). DNA integrity was verified by 1% agarose gel electrophoresis, and DNA concentration as well as purity were determined using a NanoDrop 2000 spectrophotometer (Thermo Fisher Scientific, USA). Only DNA samples with an OD_260_/OD_280_ ratio ranging from 1.8 to 2.0 were subjected to subsequent PCR amplification. Qualified DNA was amplified using bacterial 338F/806R [75] and fungal ITS1F/ITS2 [35] primer sets in a 20 µL PCR system with standard thermal cycles. Target amplicons were recovered via 2% agarose gel electrophoresis and purified. Purified amplicons were barcoded to construct libraries using the TruSeq DNA Sample Prep Kit. Library preparation and Illumina MiSeq PE300 paired-end sequencing were performed by Shanghai Majorbio Bio-pharm Technology Co., Ltd.

### Data analysis

Growth indicators, active ingredients, and soil chemical properties data were processed using the SPSS statistical software package v26.0. Prior to ANOVA, the normality of the data was evaluated using the Shapiro‑Wilk test (*P* > 0.05 as the threshold for normal distribution), and homogeneity of variances was confirmed via Levene’s test (*P* > 0.05 as the threshold for homogeneity of variances). As all data met the assumptions of normality and variance homogeneity, no data transformation was required. Each cultivation scenario was analyzed separately. One-way ANOVA was used to test the differences in the overall mean values among groups, followed by Tukey’s honestly significant difference (HSD) test for pairwise comparisons between groups. The significance level was set at *P* < 0.05.

The raw sequencing data, deposited in the Genome Sequence Archive (GSA) at the National Genomics Data Center (NGDC), China, under accession number CRA038762, were analyzed on the Majorbio Cloud Platform (https://cloud.majorbio.com). Raw paired-end reads were quality-filtered (Q < 20), merged with FLASH, and dechimerized via UCHIME. Clean tags were normalized to equal depth, singleton OTUs were removed, and remaining sequences were clustered into OTUs at 97% similarity using the UCLUST algorithm in the QIIME pipeline [19]. Taxonomic annotation was performed by aligning representative sequences of each OTU against the reference databases using the RDP Naïve Bayesian Classifier v2.13 [106], a public pre-built tool developed by the Ribosomal Database Project team. Only the official pre-trained model packaged with the software was applied, and no custom classifier retraining was performed in this study. Taxonomic assignment was executed at a confidence cutoff of 0.8; genus-unresolved OTUs were marked “unclassified”. For bacteria, the SILVA database (v138) was used; for fungi, the UNITE database (v8.2) was adopted. Specifically, Mothur software v1.48.0 [89] was used to calculate alpha diversity indices, including the Coverage index (sampling completeness); Ace, Chao, and Sobs indices (species richness); Simpson index (species dominance); and Shannon index (species diversity). The R software v3.3.1 (R Foundation for Statistical Computing, Vienna, Austria) with the vegan package v2.4-6 was applied to generate a Bray-Curtis dissimilarity matrix, and principal coordinates analysis (PCoA) was implemented using the phyloseq package v1.16.2 [69] to evaluate the similarity of microbial community structures among different samples (beta diversity). Bar charts of species composing soil microbial communities were generated simultaneously.

The nonparametric Wilcoxon rank-sum test was used to analyze the significance of differences in alpha diversity indices and microbial community taxon abundances between groups. The nonparametric PERMANOVA test was conducted to assess the significance of differences in beta diversity between groups [7]. Bar charts of growth indicators and active ingredients, alpha diversity index plots, microbial community bar charts, and species difference test plots were prepared using Origin 2021. The PCoA diagrams with 95% confidence ellipses were prepared using the ggplot2 package v2.2.1 [109] in R software.

## Results and analysis

### Responses of *G. uralensis* growth indicators and active ingredients to salt-alkali-tolerant microbial inoculant under different planting scenarios in moderately and severely saline-alkali soils

For *G. uralensis* seeds in severely saline-alkali soil, germination rates of both MI and CK groups were less than 10%. Direct sowing was unfeasible due to poor germination; thus, post-germination growth was not assessed. We therefore only studied seedling dynamics under moderate salinity (DS(M), ST(M)) and under severe salinity (ST(S)).

**Fig. 1:**
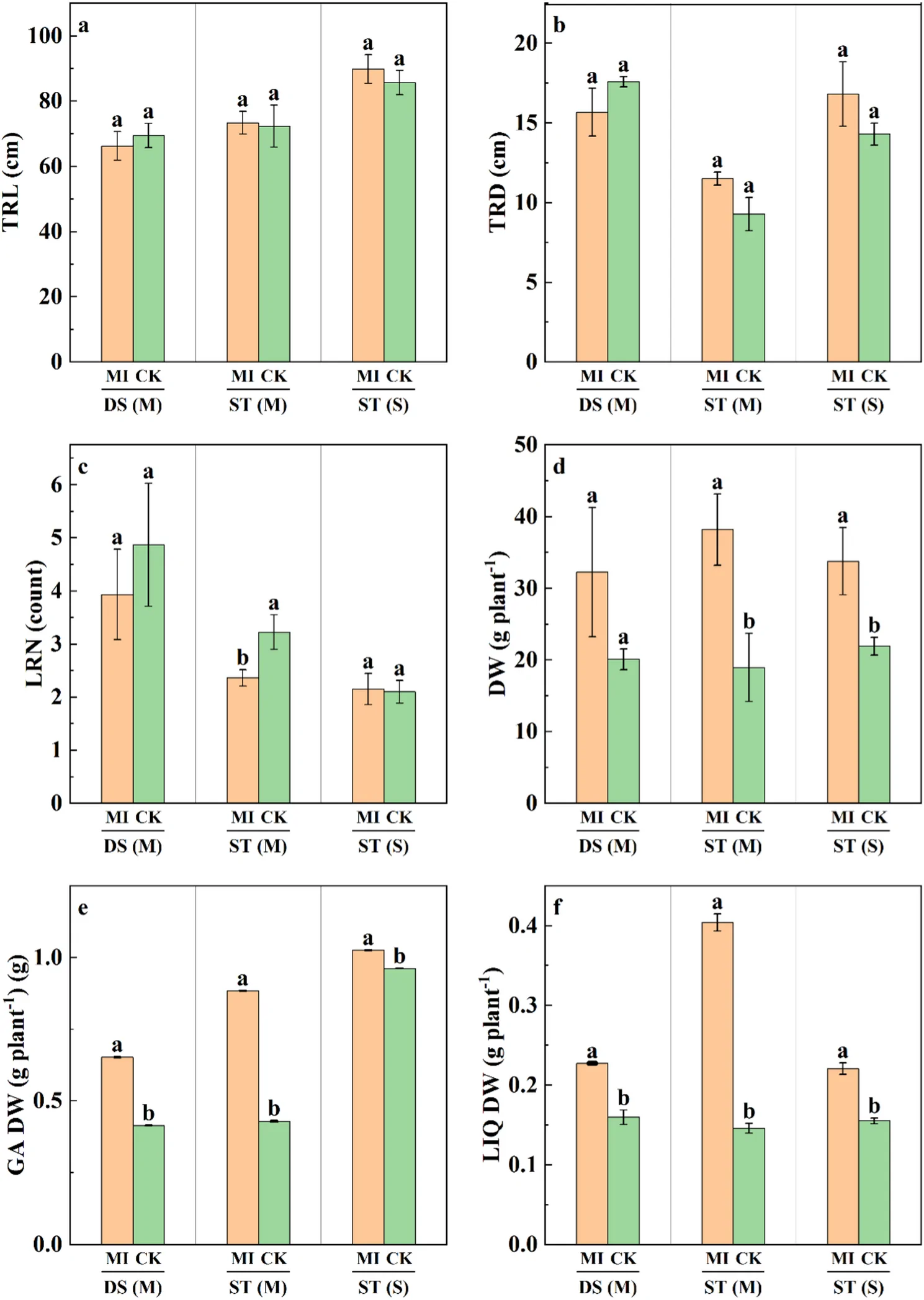
Effects of salt-alkali-tolerant microbial inoculant on growth indicators and bioactive compounds of *G. uralensis* under different scenarios in saline soils. MI: microbial inoculant group; CK: control group. Planting scenarios: DS(M), direct seeding in moderately (M) saline soil; ST(M), seedling transplant in moderately saline soil; ST(S), seedling transplant in severely (S) saline soil. **a** Taproot length (TRL), **b** taproot diameter (TRD), **c** lateral root number (LRN), **d** root dry weight (DW), **e** glycyrrhizic acid dry weight (GA DW), and **f** liquiritin dry weight (LIQ DW). Values are the mean ± SE, with different lowercase letters indicating significant differences between MI and CK (one-way ANOVA with Tukey’s post hoc test, *P* < 0.05)

The salt-alkali-tolerant microbial inoculant did not significantly affect *G. uralensis* taproot length (TRL) or taproot diameter (TRD) under the three planting scenarios (*P* > 0.05; Fig. 1a, b). A declining trend in lateral root number (LRN) was observed under ST(M) and DS(M), yet this reduction reached statistical significance only in ST(M) (*P* < 0.05).

Root dry weight (DW) of *G. uralensis* was the growth trait most responsive to the salt-tolerant microbial inoculant. MI showed a trend of higher root DW relative to CK across all three scenarios, and root DW was markedly elevated by 101.68% and 53.96% under ST(M) and ST(S).

Under all three scenarios, MI exhibited significantly higher individual dry weights of glycyrrhizic acid (GA DW) and liquiritin (LIQ DW) than the CK group (*P* < 0.05) (Fig. 1e, f). Glycyrrhizic acid contents of MI were 2.03% in DS(M), 2.11% in ST(M), and 2.94% in ST(S), while liquiritin contents were 0.72% in DS(M), 1.05% in ST(M), and 0.65% in ST(S); all contents reached the quality standards stipulated in the *Pharmacopoeia of the People’s Republic of China* (glycyrrhizic acid ≥ 2.0%; liquiritin ≥ 0.50%). Additionally, the GA DW increased by 57.32% in DS(M), 106.11% in ST(M), and 6.56% in ST(S) compared with CK, while the LIQ DW increased by 42.50% in DS(M), 177.24% in ST(M), and 42.34% in ST(S) (Fig. 1e, f).

### Responses of *G. uralensis* rhizosphere soil chemical properties to salt-alkali-tolerant microbial inoculant under different planting scenarios in moderately and severely saline-alkali soils

**Table 2:** Effects of salt-alkali-tolerant microbial inoculant on rhizosphere soil chemical properties of *G. uralensis* under three scenarios

Soil chemical properties	DS(M)		ST(M)		ST(S)	
	MI	CK	MI	CK	MI	CK
pH	7.84 ± 0.13a	7.83 ± 0.15a	8.05 ± 0.08a	7.91 ± 0.05a	7.92 ± 0.33a	7.78 ± 0.12a
SS (g kg^–1^)	7.56 ± 0.23a	7.36 ± 0.35a	7.38 ± 0.02a	7.75 ± 0.18a	9.75 ± 0.44a	9.53 ± 0.23a
TN (mg kg^–1^)	350.32 ± 7.86a	348.15 ± 3.58a	291.65 ± 1.61b	356.64 ± 2.57a	347.44 ± 4.19a	258.85 ± 23.49b
AK (mg kg^–1^)	91.68 ± 2.39b	101.62 ± 1.88a	269.37 ± 0.40a	97.42 ± 2.20b	223.32 ± 4.88b	273.69 ± 4.47a
AP (mg kg^–1^)	40.32 ± 2.04a	39.98 ± 0.85a	45.91 ± 0.39a	36.64 ± 0.60b	5.34 ± 0.09a	5.51 ± 0.18a
NO_3_^−^-N (µg g^–1^)	5.74 ± 0.58b	8.97 ± 0.85a	13.56 ± 0.54b	15.14 ± 0.34a	22.74 ± 1.06b	41.00 ± 4.22a
SOM (mg g^–1^)	5.76 ± 0.11a	5.82 ± 0.09a	4.30 ± 0.07b	6.48 ± 0.08a	7.80 ± 0.31a	6.23 ± 0.13b

Values are the mean ± standard error (SE). Different lowercase letters in the same row under individual scenarios indicate significant differences between MI and CK (one-way ANOVA with Tukey’s HSD post hoc test, *P* < 0.05). DS(M), direct seeding in moderately saline soil; ST(M), seedling transplanting in moderately saline soil; ST(S), seedling transplanting in severely saline soil. SS, soil soluble salt; SOM, soil organic matter; TN, total nitrogen; AP, available phosphorus; AK, available potassium; NO_3_^−^-N, nitrate nitrogen

Across all three scenarios, rhizosphere soil pH and SS showed no significant differences between MI and CK groups (*P* > 0.05; Table 2), while the NO_3_^−^-N content was significantly lower in MI than CK (*P* < 0.05).

TN, SOM, AK and AP exhibited inconsistent responses to microbial inoculation among the three scenarios, with significant differences between MI and CK detected in some scenarios but not in others (Table 2). For example, both TN and SOM were significantly higher in MI than CK under ST(S), whereas no significant differences in TN and SOM were detected between MI and CK under DS(M). Meanwhile, all four indicators exhibited significant between-group differences under ST(M).

### Responses of *G. uralensis* rhizosphere soil microbial α diversity indices to salt-alkali-tolerant microbial inoculant under different planting scenarios in moderately and severely saline soils

**Fig. 2:**
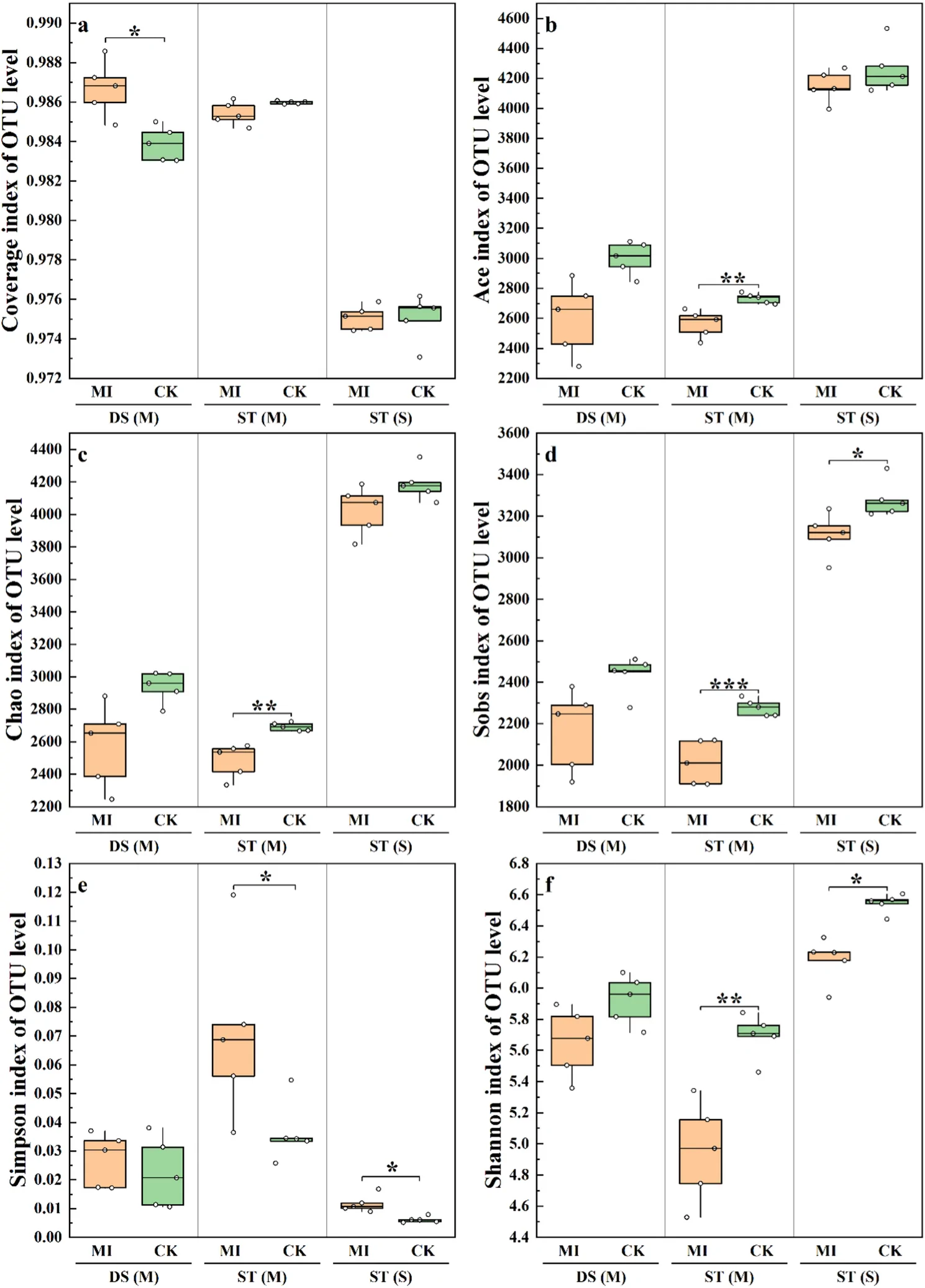
Effects of salt-alkali-tolerant microbial inoculant (MI) on α diversity indices of *G. uralensis* rhizobacteria under different scenarios. CK, control (without inoculant). Planting scenarios: DS(M), direct seeding in moderately (M) saline soil; ST(M), seedling transplanting in moderately saline soil; ST(S), seedling transplanting in severely (S) saline soil. **a** Coverage index, **b** Ace index, **c** Chao index, **d** Sobs index, **e** Simpson index, and **f** Shannon index. All indices were calculated based on operational taxonomic units (OTUs). *P < 0.05; **P < 0.01; ***P < 0.001

Across the three scenarios, the sequencing coverage index of the rhizosphere bacterial community ranged from 0.974 to 0.988, indicating sufficient sequencing depth to fully cover the species composition and reliably characterize the actual bacterial community structure (Fig. 2a).

Three species richness indices (Sobs, Chao and Ace) of rhizobacterial communities were consistently lower in MI than in CK groups (Fig. 2b-d). However, no statistically significant differences in the three indices were observed between MI and CK under DS(M). In ST(M), Ace, Chao and Sobs indices of the MI were significantly lower, demonstrating the inoculant significantly reduced rhizobacterial richness. In ST(S), only the Sobs index was significantly decreased in MI, suggesting that the inoculant exerted no significant effects on the Ace and Chao indices.

The Simpson and Shannon indices showed consistent response trends. The MI group presented higher Simpson index (Fig. 2e) and lower Shannon index (Fig. 2f) compared with the CK group. However, significant differences between MI and CK were only detected under ST(M) and ST(S).

**Fig. 3:**
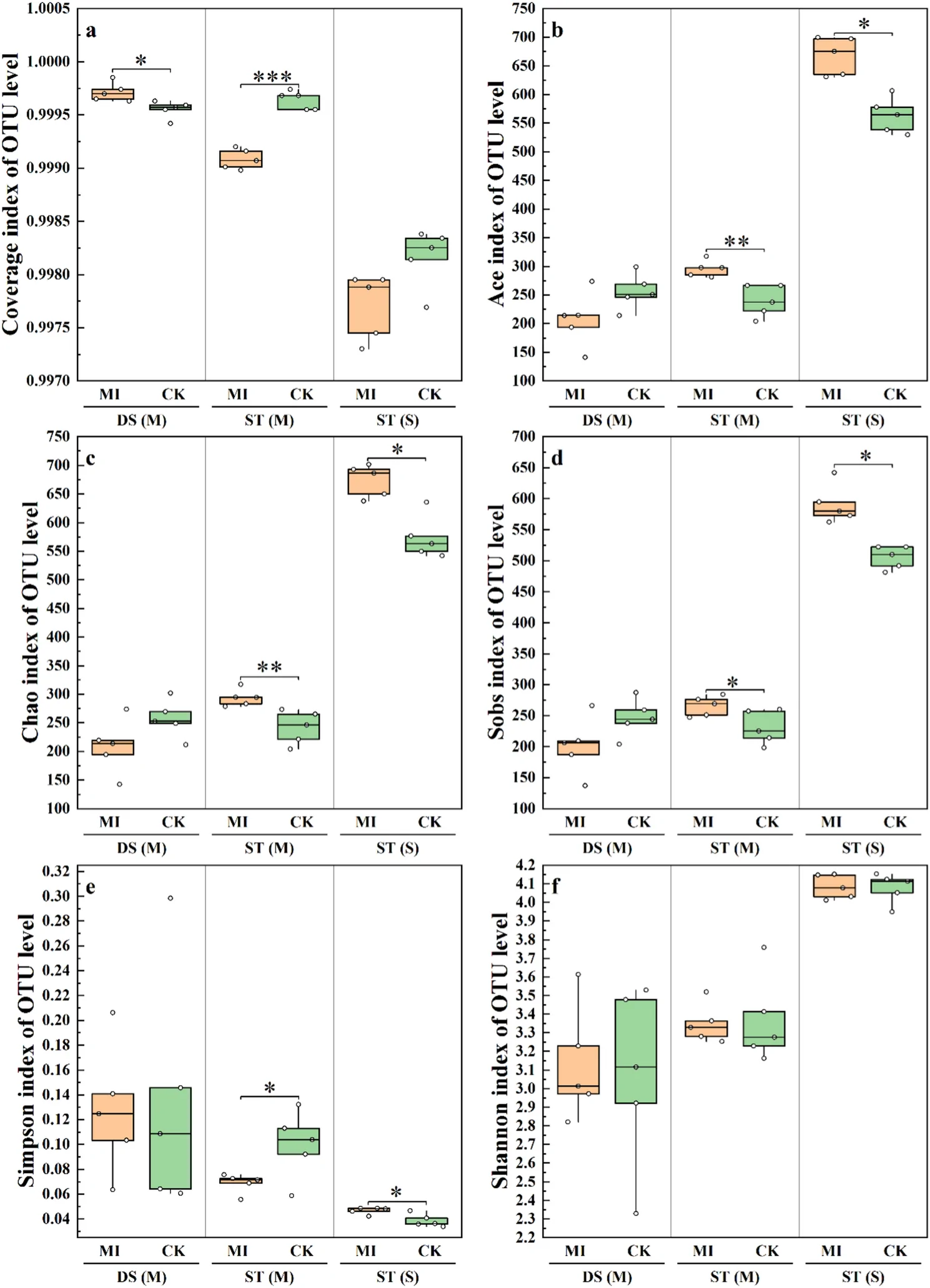
Effects of salt-alkali-tolerant microbial inoculant (MI) on α diversity indices of *G. uralensis* rhizosphere fungi under different scenarios. CK, control (without inoculant). Planting scenarios: DS(M), direct seeding in moderately (M) saline soil; ST(M), seedling transplanting in moderately saline soil; ST(S), seedling transplanting in severely (S) saline soil. **a** Coverage index, **b** Ace index, **c** Chao index, **d** Sobs index, **e** Simpson index, and **f** Shannon index. All indices were calculated based on operational taxonomic units (OTUs). *P < 0.05; **P < 0.01; ***P < 0.001

The effects of the salt-tolerant microbial inoculant on the α diversity indices of fungi in the *G. uralensis* rhizosphere are shown in Fig. 3. Across the three scenarios, the Coverage index of rhizosphere fungal communities all approached 1, indicating sufficient sequencing depth (Fig. 3a).

Across the three scenarios, no significant differences in Ace, Chao, and Sobs were detected between the MI and CK groups under DS(M). Under ST(M) and ST(S), the MI group exhibited statistically distinct increases in these indices relative to the CK group (Fig. 3b-d).

The Simpson and Shannon indices of rhizosphere fungal communities are presented in Fig. 3e-f. Consistent with the bacterial community, the inoculant exerted no significant effects on the two indices under DS(M). In contrast, the Simpson index of the MI group exhibited a significantly lower than the CK group under ST(M), whereas it was significantly higher under ST(S). However, no significant differences in the Shannon index were observed between the MI and CK groups across both ST scenarios.

### Responses of *G. uralensis* rhizosphere soil microbial β diversity to salt-alkali-tolerant microbial inoculant under different planting scenarios in moderately and severely saline soils

**Fig. 4:**
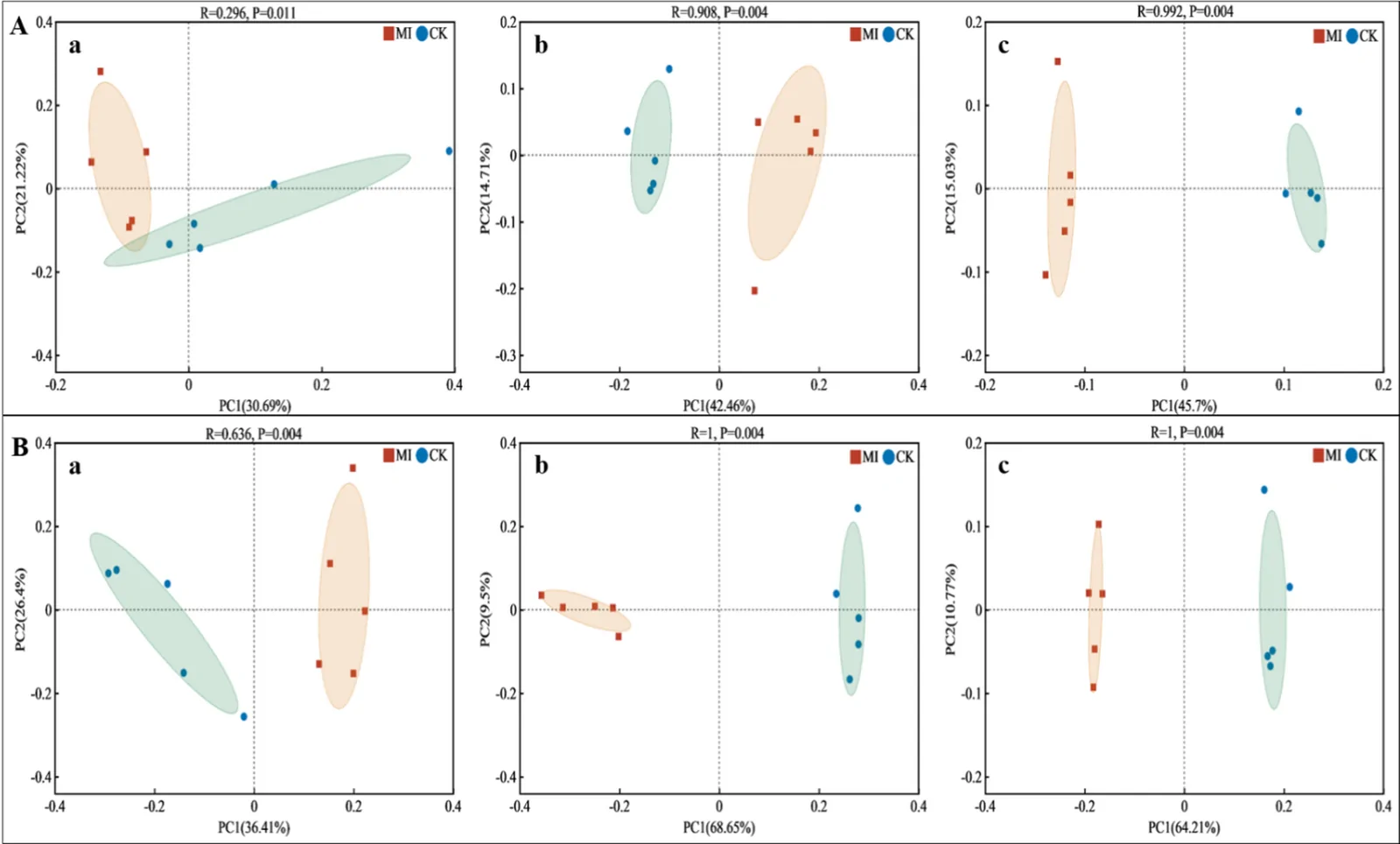
Effects of salt-alkali-tolerant microbial inoculant (MI) on β diversity of *G. uralensis* rhizosphere soil microbial communities under the three scenarios based on Bray–Curtis PCoA at the OTU level. CK, control (without inoculant). β diversity of rhizosphere (A) bacterial and (B) fungal communities under the planting scenarios **a** DS(M), direct seeding in moderately (M) saline soil; **b** ST(M), seedling transplanting in moderately saline soil; and **c** ST(S), seedling transplanting in severely (S) saline soil. Parenthetical axis values show variance explained by each principal coordinate. R ranges from 0–1, with values closer to 1 indicating greater inter-group community separation

Principal coordinate analysis (PCoA) based on Bray–Curtis dissimilarity and analysis of similarities (ANOSIM) were performed to characterize OTU-level β-diversity of rhizosphere bacterial and fungal communities of *G. uralensis* across three scenarios (Fig. 4). The 95% confidence ellipses delineating the MI and CK treatments showed clear segregation. Cumulative variances explained by PC1 and PC2 ranged from 52.91% to 60.60% for bacteria and 63.05%–78.30% for fungi, both exceeding the common 50% explanatory threshold. ANOSIM confirmed significant dissimilarity between MI and CK under all scenarios (*P* < 0.05). DS(M) exhibited the weakest community separation (bacterial *R* = 0.296, fungal *R* = 0.636), while ST(M) and ST(S) achieved complete fungal group segregation (*R* = 1, *P* = 0.004).

### Responses of dominant genera of *G. uralensis* rhizosphere soil microbial communities to salt-alkali-tolerant microbial inoculant and difference analysis of dominant genera under different planting scenarios in moderately and severely saline soils

**Fig. 5:**
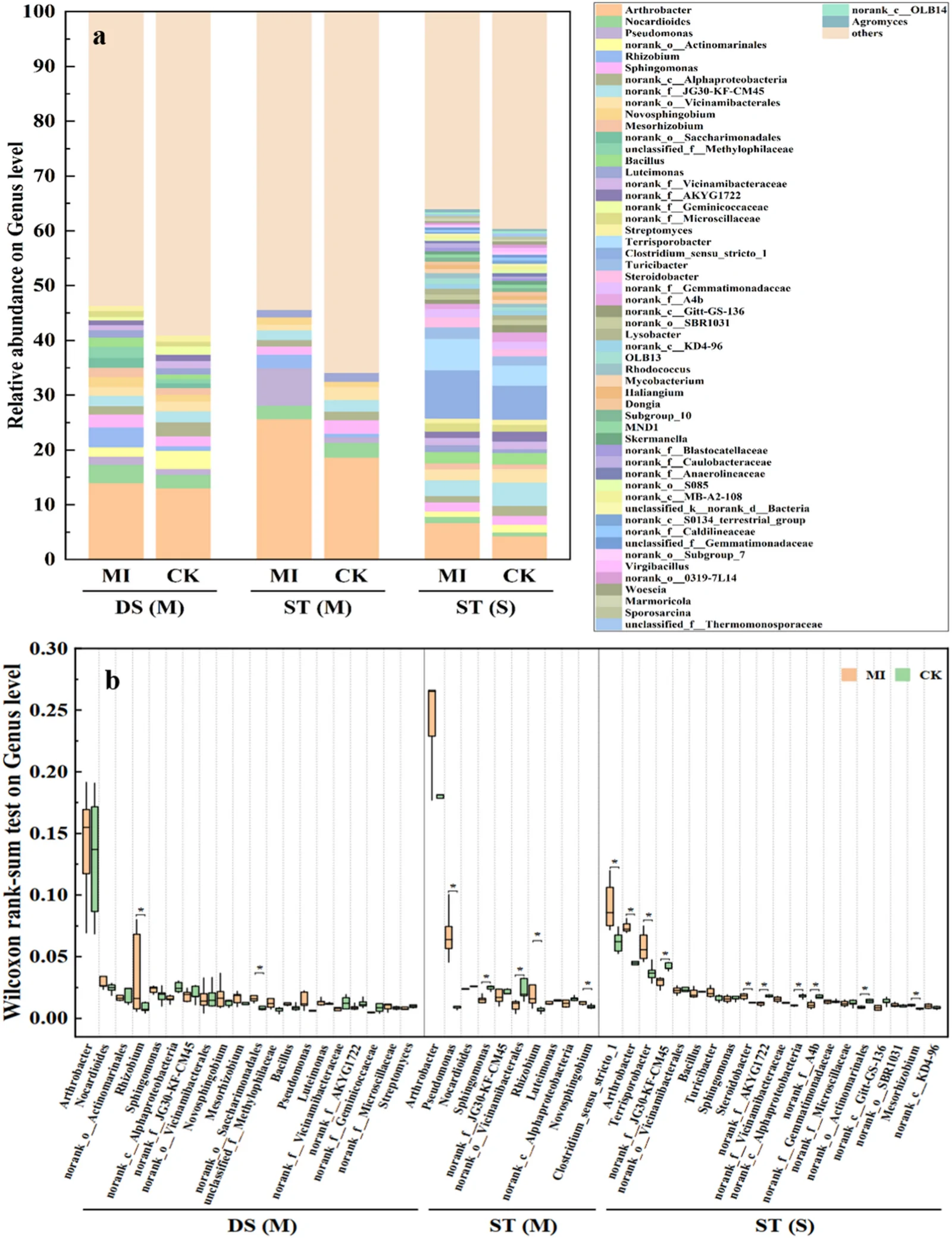
Effects of salt-alkali-tolerant microbial inoculant (MI) on the relative abundances of dominant bacterial genera in the rhizosphere of *G. uralensis* under different scenarios. CK, control (without inoculant). Planting scenarios: DS(M), direct seeding in moderately (M) saline soil; ST(M), seedling transplanting in moderately saline soil; ST(S), seedling transplanting in severely (S) saline soil. **a** Relative abundances of dominant bacterial genera; **b** differential analysis of dominant genera based on Wilcoxon rank-sum test

The dominant bacterial genera in the *G. uralensis* rhizosphere soil exhibited divergent responses to the salt-alkali-tolerant microbial inoculant across the three scenarios (Fig. 5a).

In DS(M), the three genera contained in the inoculant were among the top 20 most abundant bacterial genera. The relative abundances of *Arthrobacter*, *Rhizobium*, and *Pseudomonas* were 14.05, 3.55, and 1.44% in the MI group, compared with 13.09, 0.88, and 1.05% in the CK group, corresponding to relative increases of 7.33, 303.40, and 37.14%, respectively. Among them, *Rhizobium* showed the most prominent increase in relative abundance. Three non-inoculant genera (*Nocardioides*, *Sphingomonas*, and *Bacillus*) also displayed significant abundance increases, with increments of 36.89, 27.87, and 95.45%, respectively. Notably, *Nocardioides* and *Bacillus* exhibited over 30% higher relative abundances in MI than in CK. Wilcoxon rank-sum tests conducted under DS(M) (Fig. 5b) revealed a significant intergroup difference only in *Rhizobium*. No statistically significant differences were observed for the other five genera (*Arthrobacter*, *Pseudomonas*, *Nocardioides*, *Sphingomonas*, *Bacillus*).

In ST(M), the three genera in the inoculant were among the top 10 most abundant genera (Fig. 5a). *Arthrobacter* and *Pseudomonas* dominated the community as the two most abundant genera, with relative abundances of 25.67 and 6.82%, respectively. The relative abundance of *Rhizobium* was 2.40%. These values represented increases of 37.41, 608.33, and 263.63% relative to the CK group. Notably, two non-inoculant genera, *Sphingomonas* and *Novosphingobium*, also exhibited distinct abundance variations between the MI and CK groups. Specifically, *Sphingomonas* abundance decreased by 38.06%, while *Novosphingobium* abundance increased by 39.58%. Wilcoxon rank-sum tests indicated significant intergroup differences in the relative abundances of *Pseudomonas*, *Rhizobium*, *Sphingomonas*, and *Novosphingobium*. In contrast, no significant differences was observed for the inoculant genus *Arthrobacter* between groups (Fig. 5b).

Unlike DS(M) and ST(M), only *Arthrobacter* was among the top 50 dominant genera in ST(S), and *Pseudomonas* and *Rhizobium* were undetectable. Nevertheless, according to the Wilcoxon rank-sum tests, the relative abundance of *Arthrobacter* in the MI group (7.37%) was significantly higher than that in the CK group (4.31%), with an increase of 70.99% (Fig. 5b). Even though only *Arthrobacter* among the inoculant genera was detected, the inoculant significantly modulated the relative abundances of several dominant genera, including *Terrisporobacter*, *Steroidobacter* and *Marmoricola*. Their relative abundances increased significantly by 54.86, 46.88, and 47.22%, respectively, in MI compared with CK.

**Fig. 6:**
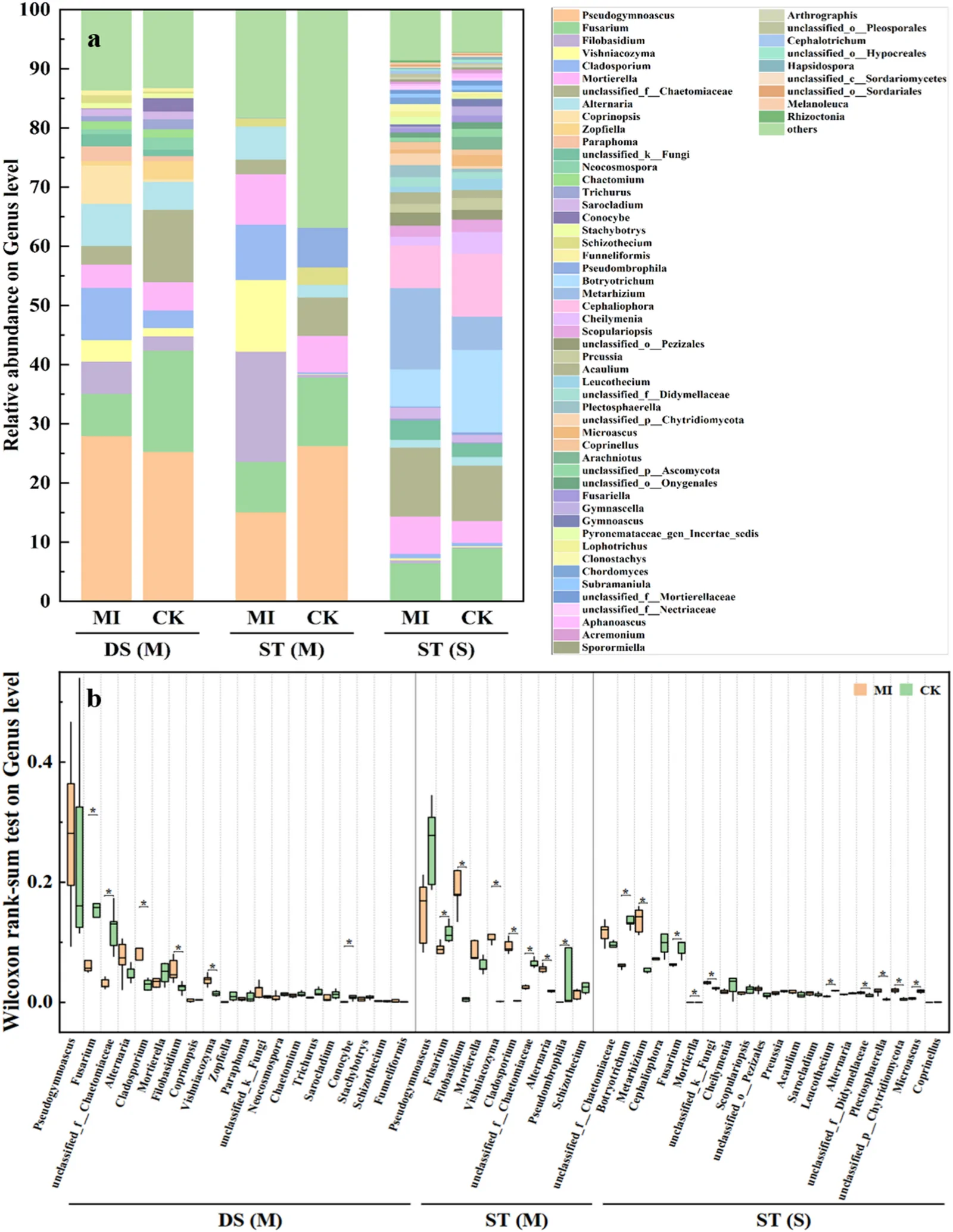
Effects of salt-alkali-tolerant microbial inoculant (MI) on the relative abundances of dominant fungal genera in the rhizosphere of *G. uralensis* under different scenarios. CK, control (without inoculant). Planting scenarios: DS(M), direct seeding in moderately (M) saline soil; ST(M), seedling transplanting in moderately saline soil; ST(S), seedling transplanting in severely (S) saline soil. **a** Relative abundances of dominant fungal genera; **b** differential analysis of dominant genera based on Wilcoxon rank-sum test. *P < 0.05

Effects of salt-alkali-tolerant microbial inoculant on the dominant fungal genera of the *G. uralensis* rhizosphere are presented in Fig. 6. The inoculant applied in this study contained no fungal strains; nevertheless, the inoculant significantly altered the relative abundances of rhizosphere dominant fungal genera across all three scenarios relative to the control (Fig. 6a).

In DS(M), among the 20 most abundant genera, the relative abundances of *Alternaria*, *Cladosporium*, *Filobasidium*, *Coprinopsis*, *Vishniacozyma*, *Paraphoma*, and *Schizothecium* were 7.18, 8.79, 5.40, 6.42, 3.70, 2.48, and 1.31% in MI, respectively, versus 4.72, 2.96, 2.41, 0.53, 1.45, 0.88, and 0.31% in CK. The relative abundance of *Alternaria* increased by 52.31%, while the remaining six genera exhibited relative abundance increases of over 100%. In contrast, *Fusarium*, *Zopfiella*, *Neocosmospora*, and *Conocybe* exhibited relative abundance reductions of 58.27, 74.83, 59.22, and 97.33%, respectively. Wilcoxon rank-sum test results showed that relative abundances of *Fusarium*, *Cladosporium*, *Filobasidium*, *Vishniacozyma*, and *Conocybe* were significantly different between MI and CK groups in DS(M) (Fig. 6b).

In ST(M), among the 10 most abundant genera of fungi, the relative abundances of *Fusarium* and *Pseudombrophila* were significantly lower in the MI group than in the CK group, according to Wilcoxon rank-sum tests, with decreases of 26.27% and 99.50%, respectively. By contrast, the relative abundances of *Filobasidium*, *Vishniacozyma*, *Cladosporium*, and *Alternaria* were significantly higher in MI than those in CK; notably, the first three displayed over 30-fold increases. Although the relative abundances of *Pseudogymnoascus* and *Schizothecium* decreased considerably and that of *Mortierella* increased markedly, those changes were not significant.

In ST(S), Wilcoxon rank-sum test results indicated that among the 20 most abundant fungal genera, the relative abundances of *Botryotrichum*, *Fusarium*, *Leucothecium*, and *Microascus* decreased significantly in MI compared with those in CK, with decreases of 55.15, 27.99, 49.63 and 66.00%, respectively. Conversely, *Metarhizium*, *Mortierella*, and *Plectosphaerella* exhibited significantly elevated relative abundances in MI relative to CK, with respective increments of 142.79, 70.14 and 306.92%. Furthermore, the relative abundance of *Cheilymenia* markedly decreased in MI relative to CK, while those of *Acaulium* and *Sarocladium* markedly increased; however, these variations were statistically non-significant.

## Discussion

This study demonstrated that salt-alkali-tolerant microbial inoculation consistently promoted root growth and bioactive compound accumulation in *G. uralensis* across three distinct cultivation scenarios, while modulating rhizosphere nutrient status and reshaping rhizosphere microbiota. Our results offer widely applicable microbially mediated amelioration strategies for multi-mode standardized *G. uralensis *cultivation in saline-alkali soilsand field evidence to develop broad-spectrum stress-resistant synthetic consortia and clarify their cross-environment stable colonization mechanisms.

### Responses of *G. uralensis* root growth indices and bioactive constituents to the microbial inoculant across three cultivation scenarios

Osmotic stress and ion toxicity induced by salt ions [8, 33, 94], adverse effects derived from seed coat damage arising from seed pretreatment [123], and elevated abundance of *Fusarium* [17] are all aggravated in severely saline-alkali soils. Collectively, these factors significantly suppress seed germination of *G. uralensis*. Seedling transplanting with fully developed root systems, endogenous nutrient reserves, and substantially greater tolerance to saline-alkali stress can circumvent the above constraints. This observation provides direct empirical support for prioritizing seedling transplantation as the cultivation strategy for *G. uralensis* in saline-alkali soils.

Across all three planting scenarios, key metrics characterizing the growth (per-plant dry biomass) and quality (active constituent concentrations) of *G. uralensis* showed uniform and significantly positive responses to saline-alkali-tolerant microbial inoculants. The inoculant-derived growth- and quality-boosting benefits did not stem from direct modifications to rhizosphere soil pH or soluble salt levels (Table 2). Instead, they were likely co-regulated by bioactive metabolites excreted by the inoculated strains as well as rhizospheric nutrient cycling and nutrient mobilization indirectly stimulated by microbial inoculation.

As mentioned previously, this inoculant is composed of *Pseudomonas silesiensis*, *Arthrobacter* sp. GCG3, and *Rhizobium* sp. DG1. All three strains exhibit diverse plant-beneficial functions, including biological nitrogen fixation, indole-3-acetic acid (IAA) secretion, and extracellular polysaccharide (EPS) production. Notably, *P. silesiensis* and *Rhizobium* sp. DG1 additionally exhibit phosphate-solubilizing and siderophore-synthesizing capacities. Furthermore, both *Arthrobacter* sp. GCG3 and *Rhizobium* sp. DG1 display antagonistic activity against the phytopathogenic fungus *Fusarium*. Zhang et al. [122].

The phytohormone IAA can directly promote the growth and development of root systems [2, 72, 123] as well as facilitate the translocation of photosynthates to sink organs, which has been verified in a wide range of plant species [10, 86, 114]. Therefore, the IAA secreted by the inoculant strains in this study may promote the development of *G. uralensis* root systems. Expanded root systems could stimulate nitrate consumption, which might account for the remarkable reduction in NO_3_^−^-N shown in Table 2. The improved nitrogen nutritional status may further activate plant carbon metabolism [58, 96], potentially supplying sufficient carbon skeletons to sustain root dry matter accumulation and the biosynthesis of bioactive secondary metabolites [27]. In addition, IAA can upregulate the catalytic activities of key rate-limiting enzymes in the secondary metabolic pathways of *G. uralensis*, such as phenylalanine ammonia-lyase and trans-cinnamate 4-monooxygenase [24, 118], thereby directly modulating the intracellular accumulation of liquiritin and indirectly leading to the de novo biosynthesis of glycyrrhizic acid [59].

Previous studies have demonstrated that the synergistic interaction between high nitrate nitrogen levels and IAA, or elevated phosphorus availability alone, can inhibit lateral root initiation [1, 99]. The moderately saline-alkali soil in this study exhibited high background AP (41.80 mg kg^−1^) and NO_3_^−^-N (48.02 µg g^−1^). Inoculation further significantly increased AP (Table 2). Under this mechanism, more photosynthates may be transported to taproots and fleshy rhizomes to curtail carbon loss allocated to lateral roots, which explains why transplanted *G. uralensis* grown in moderately saline-alkali soil displays inhibited lateral root growth and greater root dry weight.

Secondly, extracellular polysaccharides (EPS) and siderophores secreted by these strains also serve as vital bioactive substances facilitating root biomass and bioactive constituent accumulation in *G. uralensis*. Specifically, EPS can directly participate in osmoprotection and salt chelation [97] to reduce sodium uptake from the soil, enhance plant ability to take up calcium and potassium ions from the soil [4, 13], bind soil particles to form aggregates [49], stimulate biofilm formation [26], ultimately conferring salt stress tolerance to plants [3]. This functional characteristic may enable stable rhizospheric colonization [34] of inoculated strains, and the proliferating microbial populations could assimilate rhizosphere nitrate for their own growth, which may serve as an extra pathway for nitrate depletion. Meanwhile, siderophores can chelate ferric iron [85] as well as multiple essential elements including molybdenum, manganese, cobalt and nickel, and improve their utilization efficiency [6]. As an essential cofactor [87], iron can also play a vital role in glycyrrhizic acid biosynthesis [91, 92]. Overall, the regulatory processes mediated by the bioactive substances IAA, EPS, and siderophores may all provide cryptic nutrient support for *G. uralensis* growth and bioactive constituent accumulation.

SOM, AK and AP displayed distinct responses to inoculation across cultivation scenarios, with all MI–CK differences within each scenario solely driven by the inoculant. Phosphate solubilization, siderophore production, and exopolysaccharide secretion of inoculant strains may collectively mobilize soil insoluble phosphorus and mineral potassium [16, 102], which could boost the supply potential of rhizospheric AP and AK. Under the ST(M) scenario, suppressed lateral root development would directly reduce the total root absorptive surface area and lower plant acquisition of rhizospheric P and K [65, 70], potentially resulting in nutrient accumulation in the rhizosphere to a level significantly higher than that in CK. Existing studies have demonstrated that *Arthrobacter* strains secrete cellulases and lignocellulolytic enzymes [23, 95], whereas *Pseudomonas* synthesize proteases and cellulases [48, 61, 115]. We hypothesize that functional complementarity among these strains facilitates synergistic decomposition of SOM. *G. uralensis* and its rhizospheric indigenous microorganisms utilize such organic decomposition products as one nitrogen source, which exacerbates SOM depletion. While these strains replenish rhizospheric organic pools through EPS secretion and microbial necromass input, mineralization losses of native SOM surpass organic inputs derived from microbial biomass. Consequently, rhizosphere SOM concentration under MI is significantly lower relative to the control.

Inoculant application boosts the growth of *G. uralensis* under DS(M) and ST(S), as reflected by enhanced root biomass. Improved plant growth would substantially elevate whole-plant potassium demand. Nevertheless, microbially mobilized AK might fail to fulfill the potassium uptake requirement of *G. uralensis*, thereby potentially yielding markedly lower rhizosphere AK relative to the control. In severely saline soil, reduced plant metabolic activity may lower the demand for SOM. We observed a trend toward SOM accumulation in the MI group, suggesting that the rate of plant consumption was lower than the rate of SOM replenishment. We hypothesize that this accumulation is associated with SOM immobilization mediated by microbial EPS. 

Differential response mechanisms of SOM and available nutrients to inoculation across different cultivation scenarios require further targeted experimental verification.

###  Response mechanism of rhizosphere microbial communities of *G. uralensis* to the microbial inoculant across three cultivation scenarios

Variations in α diversity, β diversity, and the relative abundances of dominant genera of rhizosphere microbial communities collectively demonstrated that the microbial inoculant in this study could directionally reshape the rhizosphere microbial communities of *G. uralensis* under the three scenarios.

Inoculated strains may synchronously filter indigenous bacteria and fungi in the rhizosphere of *G. uralensis* via metabolite secretion and rhizosphere nutrient source competition [68]. Sensitive indigenous bacteria are suppressed, lowering community richness and driving the assembly of a streamlined community dominated by a small set of core dominant species [42]. Such community simplification does not signify microecological degradation but represents concentrated resource allocation, which is supported by the increased rhizosphere biomass and elevated accumulation of medicinal active ingredients. In contrast, symbiotic fungi tolerant to inoculant strains are enriched, with markedly elevated fungal community richness under moderate and severe saline-alkali transplanting scenarios. β-diversity analyses further corroborate that inoculation significantly reshapes the rhizosphere microbial community structure of *G. uralensis*. These findings also provide a clear mechanistic explanation for the more prominent variations in α-diversity within transplant groups, a pattern driven by higher intergroup community dissimilarity.

Variations in the relative abundances of dominant genera further verify such community restructuring. Within the bacterial community, besides the markedly elevated abundances of inoculated strains, certain indigenous dominant bacteria also underwent distinct shifts. For instance, under the ST(M) scenario, inoculation significantly increased the relative abundance of *Novosphingobium* by 39.58% while reducing that of *Sphingomonas* by 38.06% relative to the control. It has been reported that *Novosphingobium* can alleviate the allelopathic effects of glycyrrhizic acid on *G. uralensis* seedlings [64], degrade lignin [52] and polycyclic aromatic hydrocarbon pollutants [88], and also fix nitrogen and promote plant growth [84]. Similarly, *Sphingomonas* can produce beneficial phytohormones, remediate environmental pollutants, and ameliorate plant growth under saline-alkali stress [9]. In the present study, it is speculated that inoculant application induced shifts in the resource niches of *Novosphingobium* and *Sphingomonas*, which might contribute to reduced functional redundancy across the rhizosphere microbiome. In ST(S), only *Arthrobacter* among the inoculated strains successfully colonized and was significantly enriched, while inoculated *Pseudomonas* and *Rhizobium* were undetectable in rhizosphere sequencing data. We speculate that they either failed to establish stable colonization under high salinity, or perished post-colonization owing to suppressed metabolic activity. Nevertheless, indigenous bacteria including *Terrisporobacter*, *Steroidobacter*, and *Marmoricola* exhibited significant proliferation, which may help offset the functional deficit arising from the weak salt tolerance of the other two inoculated strains. Relevant studies have documented the functional traits of these genera. Specifically, *Terrisporobacter* degrades organic matter and humic substances [108]; *Steroidobacter* catabolizes polysaccharides [43] and steroid hormones while participating in denitrification [30]; and *Marmoricola* is positively correlated with biomass accumulation in legumes such as soybean and alfalfa [113].

Across the three planting scenarios, the relative abundance of *Fusarium*, the primary causal agent of root rot in *G. uralensis* [18, 119], decreased significantly compared with the control. The inhibition of *Fusarium* may be explained by two underlying mechanisms. First, the bacterial strains in the inoculant are directly antagonistic to *Fusarium*, a conclusion supported by our previous studies in which *Arthrobacter* sp. GCG3 and *Rhizobium* sp. DG1 significantly inhibited the pathogen [121, 122]. Second, the inoculant indirectly suppresses pathogenic fungi by increasing the competitive advantage of indigenous bacteria and fungi. For example, *Cladosporium* and *Vishniacozyma*, which have been documented to possess disease-suppressive traits [37, 44, 77], were significantly enriched under DS(M) and ST(M). In ST(S), *Mortierella* and *Metarhizium*, which were significantly enriched, are widely recognized as fungi with plant biocontrol and growth-promoting functions [29, 45, 60, 107]. In addition, the significant enrichment of the indigenous bacterial genus *Marmoricola* under ST(S) is consistent with earlier research reporting a negative correlation between this genus and *Fusarium* [39]. The uniform reduction in *Fusarium* abundance across the three cultivation scenarios directly lowered the incidence of root diseases in *G. uralensis*, thereby allowing healthy roots to support biomass accumulation.

Notably, certain fungal genera have been reported to possess dual capacities of pathogenic infection and organic matter degradation. For instance, *Alternaria* [90, 103], which was significantly enriched under ST(M), and *Plectosphaerella* [20, 74], a genus that also exhibited remarkable enrichment under ST(S), yet neither induced disease symptoms in *G. uralensis*. In this study, we hypothesize that inoculant application may enhance their fitness within the rhizosphere of *G. uralensis*. These fungi may interact synergistically with other fungal taxa to exert beneficial effects, promoting both plant growth and secondary metabolite accumulation.

Collectively, these results corroborate that the seedling transplanting mode is more compatible with the functional inoculant, producing pronounced intergroup differences in rhizosphere microbiota. By contrast, although shifts in α-diversity and the relative abundance of dominant genera were observed under direct seeding in DS(M), intergroup differences were largely non-significant. Nevertheless, the significant separation in overall community structure reflected by β-diversity indices still supported the conclusion that inoculation reshapes the rhizosphere microbial community of *G. uralensis* under DS(M).

**Fig. 7:**
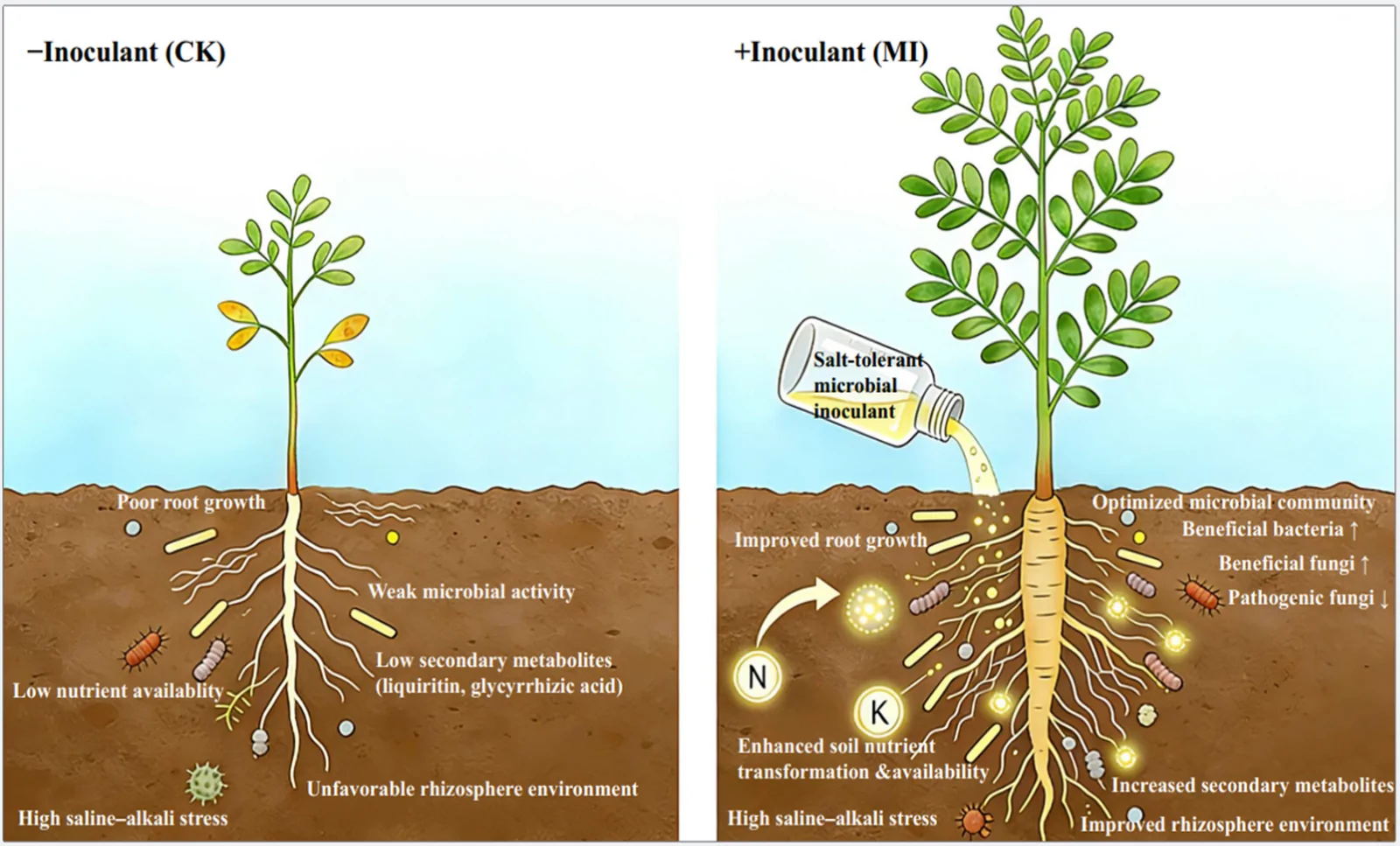
Conceptual model illustrating the effects of the salt-alkali-tolerant microbial inoculant on the rhizosphere processes of *G. uralensis*

The potential mechanisms underlying the beneficial effects of the salt-alkali-tolerant microbial inoculant are summarized and illustrated in Fig. 7.

## Conclusions

In this study, we carried out a field experiment in saline-alkali soils of Lanzhou, Gansu Province, Northwest China with distinct cultivation scenarios to investigate the effects of a salt-alkali-tolerant microbial inoculant on root growth, bioactive constituent accumulation, rhizosphere nutrient conditions, and rhizosphere microbial communities of *G. uralensis*. Across all cultivation scenarios, the microbial inoculant consistently promoted root development and the accumulation of bioactive compounds in *G. uralensis*, while regulating rhizosphere soil nutrients and reshaping the structural composition of rhizosphere microbial communities.

Our findings confirm the effectiveness of salt-alkali-tolerant plant growth-promoting microorganisms in mitigating growth suppression of medicinal plants under saline-alkali stress. Beyond presenting a feasible biological regulation approach to remediate saline-alkali degraded soils, this study lays a theoretical foundation for developing customized microbial inoculants suited to various saline-alkali soil types. Furthermore, this work provides valuable theoretical insights into microbial regulation research for other medicinal plants cultivated in saline-alkali areas. Most notably, we revealed that the combined use of microbial inoculation and seedling transplanting represents a suitable cultivation strategy for *G. uralensis* grown in moderately to severely saline-alkali soils, since this planting pattern enables plants to fully tap into the regulatory advantages of microbial inoculants. 

We also acknowledge several limitations of this study. First, the short experimental period cannot reflect the long-term succession patterns of microbial communities after inoculant amendment. Second, the molecular and physiological pathways that underpin inoculant efficacy require further experimental validation. Future work will set up long-term stationary field trials to monitor community temporal dynamics, and combine multi-omics profiling with in vitro and pot tests to systematically decode the core functional pathways of the microbial agent. These work will lay a solid theoretical foundation for targeted optimization and efficient field deployment of such bioinoculants. From the perspective of field practical application, sustained financial support is needed for multi-year saline-alkali field trials to validate the persistent efficacy of this composite agent. Meanwhile, region-specific technical protocols combining seedling transplantation and microbial inoculation can be formulated to standardize large-scale licorice planting in arid saline soils.

## Data Availability

Raw sequencing data were deposited in the Genome Sequence Archive (GSA) at the National Genomics Data Center (NGDC), China, under accession number CRA038762 and are available at the following URL: https://download.cncb.ac.cn/gsa6/CRA038762.
